# Highly responsive MoS_2_ photodetectors enhanced by graphene quantum dots

**DOI:** 10.1038/srep11830

**Published:** 2015-07-03

**Authors:** Caiyun Chen, Hong Qiao, Shenghuang Lin, Chi Man Luk, Yan Liu, Zaiquan Xu, Jingchao Song, Yunzhou Xue, Delong Li, Jian Yuan, Wenzhi Yu, Chunxu Pan, Shu Ping Lau, Qiaoliang Bao

**Affiliations:** 1 Institute of Functional Nano and Soft Materials (FUNSOM), Jiangsu Key Laboratory for Carbon-Based Functional Materials and Devices, and Collaborative Innovation Center of Suzhou Nano Science and Technology, Soochow University, Suzhou 215123, P. R. China; 2 Department of Applied Physics, The Hong Kong Polytechnic University, Hung Hom, Hong Kong SAR, China; 3 Department of Materials Engineering, Monash University, Clayton, VIC 3800, Australia; 4 School of Physical and Technology, Wuhan University, Wuhan 430072, P. R. China

## Abstract

Molybdenum disulphide (MoS_2_), which is a typical semiconductor from the family of layered transition metal dichalcogenides (TMDs), is an attractive material for optoelectronic and photodetection applications because of its tunable bandgap and high quantum luminescence efficiency. Although a high photoresponsivity of 880–2000 AW^−1^ and photogain up to 5000 have been demonstrated in MoS_2_-based photodetectors, the light absorption and gain mechanisms are two fundamental issues preventing these materials from further improvement. In addition, it is still debated whether monolayer or multilayer MoS_2_ could deliver better performance. Here, we demonstrate a photoresponsivity of approximately 10^4^ AW^−1^ and a photogain of approximately 10^7^ electrons per photon in an *n-n* heterostructure photodetector that consists of a multilayer MoS_2_ thin film covered with a thin layer of graphene quantum dots (GQDs). The enhanced light-matter interaction results from effective charge transfer and the re-absorption of photons, leading to enhanced light absorption and the creation of electron-hole pairs. It is feasible to scale up the device and obtain a fast response, thus making it one step closer to practical applications.

Layered transition metal dichalcogenides (TMDs, MX_2_, M = Mo, W, Nb, Ta, Ti, Re; X = S, Se, Te) have attracted increasing attention as channel materials for field-effect transistors (FETs) because of their high electron mobility, excellent current ON/OFF ratio and low subthreshold swing^1^,^2^,^3^,^4^,^5^,^6^. As a typical semiconductor from the family of TMDs, MoS_2_ has a tunable bandgap that varies with thickness; that is, bulk MoS_2_ has an indirect bandgap of 1.2 eV and, because of quantum confinement, monolayer MoS_2_ has a direct bandgap of 1.8 eV^1^,^2^,^3^. Atomically thin films of MoS_2_ have excellent photoactive properties in terms of strong resonant light absorption (>20%) and high quantum luminescence efficiency^4^,^7^, which makes these materials ideal for optoelectronic applications such as light emitting diodes^8^,^9^ and photodetectors^10^,^11^,^12^,^13^,^14^,^15^.

The first phototransistor based on a mechanically exfoliated MoS_2_ monolayer showed a photoresponsivity of only 7.5 mAW^−1^ (Ref. 13) which is comparable to that of graphene-based devices (6.1 mAW^−1^)^16^. The performance of phototransistor based on monolayer MoS_2_ can be improved significantly because it possesses a high carrier mobility (up to approximately 410 cm^2^V^−1^s^−1^)^17^ and strong resonant absorption. By optimising the fabrication technique, photodetectors based on mechanically exfoliated monolayer MoS_2_ can achieve an impressively high responsivity of 880 AW^−1^ (Ref. 10). It should be noted, however, that multilayer MoS_2_ has a density of state that is three times higher than monolayer MoS_2_^18^, as well as a wider spectrum response^12^ because of its narrower bandgap, which could afford effective broadband detection. Because it is still challenging to grow large area monolayer MoS_2_ via chemical vapour deposition (CVD), the use of multilayer MoS_2_ films as the channel material in phototransistors is likely to be a more practical solution, mainly because the growth of large area multilayer MoS_2_ films on different substrates is quite successful^19^,^20^,^21^,^22^. Nevertheless, the indirect bandgap of multilayer MoS_2_ film intrinsically limits the photoresponsivity and photogain of such photodetectors.

In this work, we hybridised a multilayer MoS_2_ film with GQDs to create a new channel material for photodetection where the GQDs function as an effective gain material. High quality and large area multilayer MoS_2_ films were grown on SiO_2_/Si substrates by the reaction of gaseous MoO_3_ and sulphur via atmospheric pressure chemical vapour deposition (APCVD). This growth technique affords a simple device fabrication process that does not require material transfer. Because of fewer defects and impurities, multilayer MoS_2_ films demonstrate surprisingly good performance for photodetection, which is comparable to the best record achieved on mechanically exfoliated MoS_2_^10^.

To explore the performance limit of this material, we modified the MoS_2_ thin film by incorporating nitrogen-doped graphene quantum dots (GQD_S_), which possess strong light absorption, long carrier lifetime and a sizable bandgap upon excitation^23^,^24^,^25^,^26^. An *n-n* type van der Waals heterostructure consisting of GQDs and MoS_2_ is formed, where the photoexcited holes can be induced successfully by MoS_2_ and are subsequently trapped because of a small energy barrier in the heterojunction. The long carrier lifetime of the GQDs will cause the photoexcited carriers to recirculate several times at the interface. Moreover, light emission from the GQDs will be re-absorbed by MoS_2_ to generate additional photocarriers. Consequently, the MoS_2_-GQDs hybrid system is an extremely efficient photoelectrical material that can achieve impressive photodetection performance. Indeed, this material ushers in an innovative approach for fabricating highly responsive and low cost photodetectors for practical applications.

## Results and Discussion

**Figure 1:**
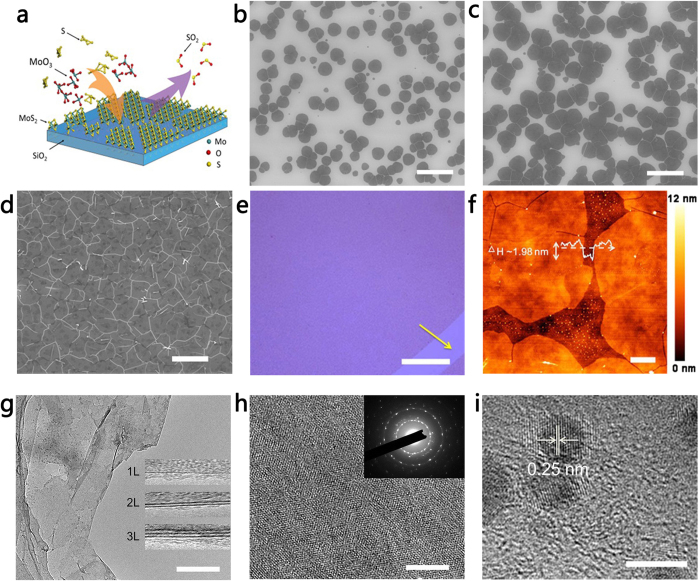
Material characterisations of MoS_2_and GQDs. (**a**) Schematic diagram showing the chemical reaction on a SiO_2_ substrate for the production of MoS_2_. (**b–d**) SEM images showing different growth stages of MoS_2_ with reaction times of 5, 10 and 30 min, respectively. Scale bars in (**b–d**): 20 μm. (**e**) Optical image of MoS_2_ on a SiO_2_ substrate. The yellow arrow indicates a scratch. Scale bar: 50 μm. (**f**) AFM topography of a MoS_2_ film on a SiO_2_ substrate. The white profile indicates three atomic layers. Scale bar: 1 μm. (**g**) TEM image showing a folded MoS_2_ film. Scale bar: 200 nm. The inset shows the folded edges of monolayer (1L), bilayer (2L) and trilayer (3L) MoS_2_ films. (**h**) HRTEM image of a MoS_2_ film. The scale bar is 5 nm. The inset shows the corresponding electron diffraction pattern. (**i**) HRTEM image of GQDs. Scale bar: 2 nm.

We use the CVD method to controllably synthesise large area MoS_2_ films on SiO_2_/Si substrates. Details of the experimental setup and growth process are provided in the Methods and Supporting Information (Fig. S1). Figure 1a schematically illustrates the chemical reaction on a SiO_2_ substrate for the production of MoS_2_. Briefly, MoO_3_ is largely reduced by sulphur vapour to form MoO_3-x_ species, which are further sulphurised and accompanied by the formation of MoS_2_ on the SiO_2_ substrate^7^,^21^. The growth of MoS_2_ follows a typical two-dimensional growth mode consisting of nucleation, growth and coalescence, as shown in Fig. 1b–d. The dark contrast in the images corresponds to MoS_2_ crystals, and the surrounding area with bright contrast is the SiO_2_ substrate. It is found that shorter growth times (*e.g.*, 5 min, Fig. 1b) will produce several atomically thin MoS_2_ crystals with lateral sizes of 1–3 μm. Prolonging the growth time leads to an increase of nuclei density and a thicker layer. As a result, the newly grown MoS_2_ crystals grow larger and coalesce with each other (Fig. 1c). Eventually, a continuous film is formed with an even longer growth time (*e.g.*, 30 min, Fig. 1d). The uniform contrast in the optical image (Fig. 1e) over a large area suggests that the MoS_2_ thin film is continuous and uniform.

In the MoS_2_ layered structures, each layer has a thickness of 6–7 Å^1^,^27^. It is found that our samples are predominately 1–3 atomic layers thick. Atomic force microscopy (AFM) images (Fig. 1f) further confirm the number of layers of the MoS_2_ film, in which the thickness of the MoS_2_ trilayer is measured to be approximately 1.98 nm. Further characterisation of the thickness of monolayer and bilayer MoS_2_ is presented in the Supporting Information (Fig. S2). Figure 1g shows a transmission electron microscopy (TEM) image of a MoS_2_ film. The number of layers in the MoS_2_ film can be determined by counting the dark fringes of the folded edges (inset of Fig. 1g). High-resolution transmission electron microscopy (HRTEM) is also employed to reveal the atomic lattice of the MoS_2_ film, as shown in Fig. 1h. The selected area electron diffraction (SAED) pattern (inset of Fig. 1h) shows multiple six-fold symmetry spots, indicating that the MoS_2_ film contains several grains possessing different crystal orientations. The HRTEM image of the GQDs (Fig. 1i) shows that the size of the GQDs is approximately 3–5 nm, where the in-plane lattice with a space of 0.25 nm is clearly resolved^25^.

**Figure 2:**
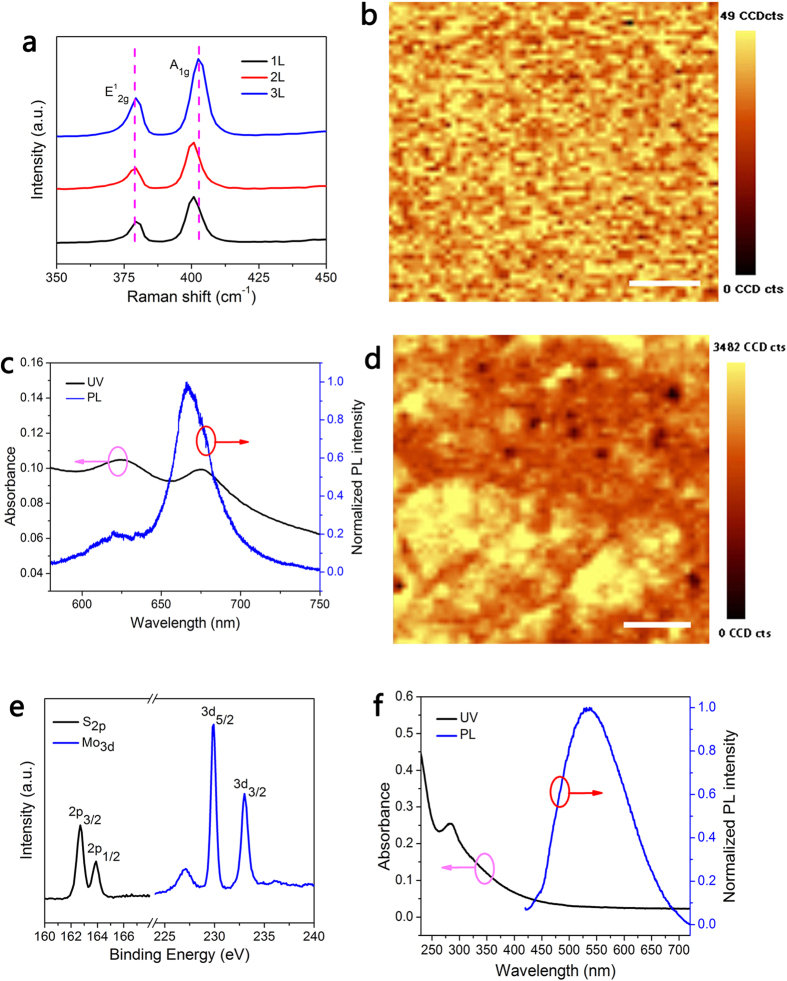
Spectroscopic characterisations of MoS_2_ films. (**a**) Raman spectra of as-grown monolayer (1L), bilayer (2L) and trilayer (3L) MoS_2_ on SiO_2_/Si substrates. (**b**) Raman image of the characteristic peak (*A*_1g_) integrated from 395 to 410 cm^−1^. Scale bar: 4 μm. (**c**) PL and UV-visible spectra of a multilayer MoS_2_ film. (**d**) PL image of the characteristic peak at 670 nm obtained by integrating from 610 to 710 nm. Scale bar: 4 μm. (**e**) X-ray photoelectron spectroscopy (XPS) spectra of a thin MoS_2_ film. (**f**) PL and UV-visible spectra of GQDs.

Spectroscopic characterisations are performed to systematically study the quality and uniformity of the MoS_2_ film. Figure 2a shows the Raman spectra obtained from different locations on as-grown MoS_2_ films with different thicknesses. Two characteristic peaks, which correspond to the *E*^1^_2g_ and *A*_1g_ vibrational modes^28^,^29^,^30^, are observed. The difference (Δ) between these modes in the as-synthesised MoS_2_ monolayer is approximately 20.6 cm^−1^, which is slightly larger than the mechanically exfoliated monolayer MoS_2_ because of crystalline imperfections. The peak differences (Δ) of bilayer and trilayer MoS_2_ are 21.4 and 23.1 cm^−1^, respectively, which are consistent with a previous report^29^. As the number of layers increases, it is found that the *E*^1^_2g_ peak redshifts whereas the *A*_1g_ peak blueshifts obviously. This observation is attributed to columbic interactions and possible stacking-induced changes in the intra-layer bonding^28^. Additionally, we also captured the Raman image of MoS_2_ by integrating the *A*_1g_ peak to verify the thickness uniformity, as shown in Fig. 2b. This figure clearly reveals that the intensity of the *A*_1g_ peak, which scales linearly with the thickness, is homogeneous over the entire scanning area (approximately 20 μm).

Figure 2c shows the UV-visible absorption and photoluminescence (PL) spectra of the MoS_2_ film. Two absorption peaks at approximately 620 nm and 670 nm are observed, which correspond to direct excitonic transitions at the Brillouin zone *K* point in MoS_2_^4^. Correspondingly, the PL spectrum depicts two feature peaks located at 620 nm and 670 nm, respectively. The PL mapping result (Fig. 2d) suggests that the MoS_2_ film is relatively uniform. The X-ray photoelectron spectroscopy (XPS) result in Fig. 2e clearly depicts the Mo-related peaks (*i.e.*, Mo-3*d*_3/2_ at 232.9 eV and Mo-3*d*_5/2_ at 229.9 eV) and the S-related peaks (*i.e.*, S-2*p*_1/2_ at 163.9 eV and S-2*p*_3/2_ at 162.7 eV), which are consistent with the reported values for MoS_2_ crystals^20^,^31^. If we compare the integration area of these two sets of peaks, we are able to conclude that the atomic ratio of Mo and S is close to 1:2. Figure 2f shows the UV-visible absorption and PL spectra of GQDs. It is found that the GQDs possess an absorption peak at 283 nm and a broad PL peak from 450 nm to 700 nm. These results are similar to previously published reports^24^,^25^.

**Figure 3:**
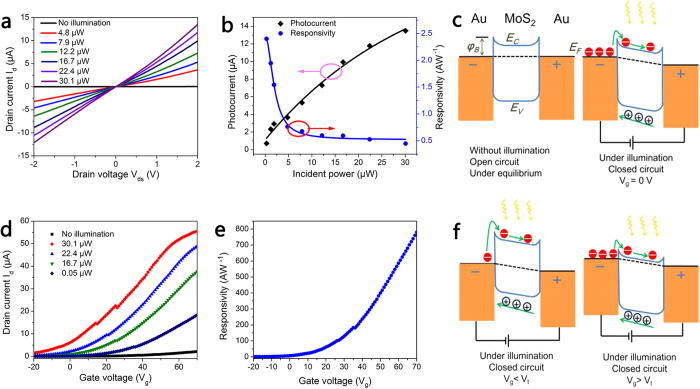
Optoelectronic characterisations of a multilayer MoS_2_ phototransistor. (**a**) Device drain current (*I*_d_) as a function of source-drain voltage (*V*_ds_) for an excitation wavelength of 405 nm with different incident powers. (**b**) The dependence of photocurrent and photoresponsivity on incident power. The blue and black dots are the original data, whereas the lines are the exponential curves. (**c**) Schematic energy diagram of the device without and with illumination showing the energy barrier and photocurrent generation process. There is a non-zero source-drain bias (*V*_ds_ ≠ 0 V), but the gate bias is kept at zero (*V*_g_ = 0 V). (**d**) Device drain current as a function of gate voltage under different illumination powers. Source-drain bias: *V*_ds_ = 1 V. (**e**) The photoresponsivity versus back gate voltage. Source-drain bias: *V*_ds_ = 1 V; light power: 50 nW. (**f**) Schematic energy diagram showing photocurrent generation processes with different gate bias (*V*g < V_t_ and *V*_g_ > *V*_t_). There is a non-zero source-drain bias.

Figure 3a depicts the device photoresponse modulated by light power at an excitation wavelength of 405 nm. We can clearly see that the device drain current (*I*_d_) demonstrates enhancement under light illumination and that it increases at higher light power. At an excitation power of 30.1 μW, the device drain current reaches 10.15 μA when the source-drain voltage *V*_ds_ = 1.68 V, which is 1000 times higher than the current in the dark state (*i.e.*, at 11 nA). Such a high optical ON-OFF ratio is comparable to the highest ratio reported for single or multilayer MoS_2_ phototransistors^10^,^12^. Moreover, our device demonstrates high sensitivity that is responsive to very weak light (5 nW, Fig. S4). However, the device response is quite slow (with rise and decay times longer than 50 s). This slow response is likely caused by either defects or charge impurity states inside the bandgap or by the presence of trap states between MoS_2_ and the underlying SiO_2_ layer, which usually occurs for MoS_2_ grown with the CVD method^10^,^11^,^32^.

Figure 3b shows the corresponding photocurrent and photoresponsivity curves as functions of incident power. It is noteworthy that the photocurrent increases exponentially while prompting the light power, whereas the responsivity drops exponentially. whereas the responsivity drops exponentially. This effect might be related to the multifaceted generation, separation and transport processes of photoexcited carriers^33^, and might also be caused by the trap states inside multilayer MoS_2_ or between MoS_2_ and the SiO_2_ substrates^10^,^11^,^32^. The photocurrent generation can be explained by the energy diagram in Fig. 3c. Here, a typical metal-semiconductor contact between MoS_2_ and the Au electrodes indicates the band bending at the interface. An energy barrier hinders the carrier injection from the electrodes under dark conditions. However, electrons inside the valence band (*VB*) can be easily excited to the conduction band (*CB*) by photons when the device is biased and exposed to incident light, which will greatly increase the concentration of transported carriers.

The multilayer MoS_2_ phototransistor has good gate tunability, which shows higher drain current at higher gating voltage, as shown in Fig. 3d. These results clearly show that the drain currents of both the OFF and ON states increase dramatically under light illumination. With a positive gate voltage (*e.g.*, *V*_g_ = 70 V), the device can generate a photocurrent as high as 16.2 μA, even at a very low excitation light power of 50 nW. The photoresponsivity is estimated to be on the order of 10^2^ AW^−1^ while the gate bias is applied, as shown in Fig. 3e. The highest photoresponsivity is 800 AW^−1^ (*V*_g_ = 70 V), which is comparable to the best reported performance of a phototransistor based on monolayer or multilayer MoS_2_^10^,^12^,^13^,^15^. The gate modulated photocurrent can be well understood by the energy diagram in Fig. 3f. Depending on the relationship between the gate bias (*V*_g_) and the threshold voltage (*V*_t_), the Fermi level in MoS_2_ can be shifted up (*V*_g_ > *V*_t_) or down (*V*_g_ < *V*_t_) while sweeping the back gate voltage, which results in the ON or OFF switch of the phototransistor.

**Figure 4:**
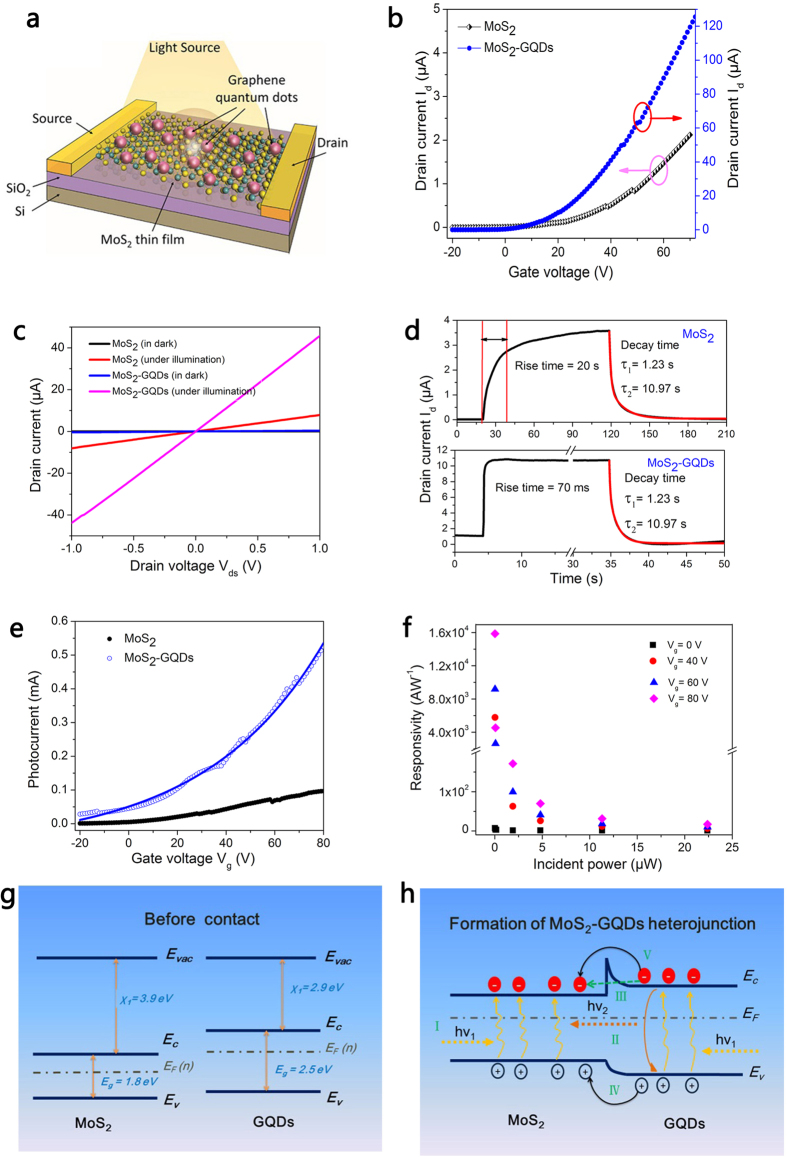
Optoelectronic characterisations of a MoS_2_-GQDs phototransistor. (**a**) Schematic of a MoS_2_-GQDs heterostructure phototransistor. (**b**) Typical transfer curves of MoS_2_ and MoS_2_-GQDs transistor devices. *I*_*d*_: drain current. Source-drain voltage: *V*_ds_ = 1V. (**c**) Drain current (*I*_d_) as a function of source-drain voltage (*V*_ds_) without and with light illumination for MoS_2_ and MoS_2_-GQDs phototransistors. Laser wavelength: 405 nm; light power: 17 μW. (**d**) Time-dependent photoresponse of MoS_2_ (top) and MoS_2_-GQDs (bottom) devices. (**e**) Photocurrent as a function of back gate voltage for MoS_2_ and MoS_2_-GQDs devices. Incident light power: 30.1 μW. The blue and black dots are the original data; the blue line is included for clarity. (**f**) The dependence of photoresponsivity on incident light power under back gate modulation. (**g**) Energy diagram of MoS_2_ and GQDs before contact. (**h**) Energy diagram of the interface between MoS_2_ and GQDs after the formation of a heterojunction. Five photoelectrical processes are proposed: I, Photon excitation in MoS_2_ and the GQDs; II, Re-absorption of the emitted photons from the GQDs by MoS_2_; III, Electrons tunnelling from the GQDs to MoS_2_; IV, Hole transfer from the GQDs to MoS_2;_ V: Thermal excitation of electrons from the GQDs to MoS_2_.

With the aim to further increase the photoresponsivity and shorten the response time, *n*-doped GQDs were hybridised with MoS_2_ to form a heterostructure device, as shown in Fig. 4a. The dramatic change in the transfer characteristics of the MoS_2_-GQDs phototransistor is noteworthy, as shown in Fig. 4b. The threshold voltage shifts to a lower voltage and the drain current increases by 60 times for the ON state. From the *I*-*V* characterisations shown in Fig. 4c, we can see that the MoS_2_-GQDs phototransistor has a much larger drain current under illumination, which is approximately 5 times higher than a pure MoS_2_-based phototransistor. Time-dependent photoresponse measurements have also been performed, as shown in Fig. 4d. Here, we can see that the device rise time is significantly reduced from 20 s to 70 ms following the incorporation of GQDs. It is proposed that the drain current increasing rate (Δ*I*_*ph*_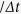) is caused by the increased carrier concentration. Another interesting phenomenon is that the fall times of the drain current, both τ_1_ and τ_2_, are reduced by a factor of ten. It is generally believed that the fast decay time component τ_1_ is related to the direct recombination of photoexcited carriers and that the slow decay time component τ_2_ is caused by sub-bandgap emissions resulting from the charge impurity and trap states inside the bandgap of MoS_2_^11^,^32^. The greatly shortened decay time is related to the efficient charge transfer between MoS_2_ and the GQDs.

Relative to a MoS_2_-based phototransistor, the MoS_2_-GQDs phototransistor has a superior gate tunability in terms of higher photocurrent, as shown in Fig. 4e. The photocurrent of our MoS_2_-GQDs device can be effectively modulated by the back gate voltage, and the photocurrent value can reach as high as 0.55 mA under an adequate gate bias (*V*_g_ = 80 V), which is 5 times that of a MoS_2_ device. Figure 4f shows the dependence of device photoresponsivity on incident light power under different back gate biases. Similar to a thin film MoS_2_ device, the responsivity decreases exponentially as the light power increases, and the maximum responsivity is 1.6 × 10^4^ AW^−1^ under an excitation power of 50 nW (*V*_g_ = 80 V), which is two orders of magnitude higher than a pure MoS_2_ device, and among the highest responsivity recorded for MoS_2_ photodetectors^10^,^12^.

To further evaluate the performance of the device, we calculated the photoconductive gain using 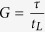, where *τ* is the carrier life time extracted from the current decay curve (Fig. 4d, τ = τ_2_ = 1.10 s) and *t*_*L*_ is the carrier transit time. The carrier transit time can be calculated by 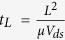, where *L* is the channel length (*L* = 20 μm for our devices), *μ* is field effect carrier mobility and *V*_ds_ is source-drain voltage. The carrier mobility 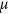 can be extracted from the device transfer curve shown in Fig. 4b using the equation 
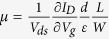
, where *ε* is the dielectric constant of SiO_2_, *d* is the thickness of SiO_2_ (d = 300 nm), and *W* is the channel width. Accordingly, we can determine a photoconductive gain up to approximately 2.4 × 10^7^, which is comparable to other reported atomically thin graphene-MoS_2_ heterostructure phototransistors^34^,^35^ and graphene-QDs hybrid photodetectors^33^.

To understand the influence of GQDs on the MoS_2_ device and the mechanism of photocurrent generation, we propose a schematic energy band diagram, as illustrated in Fig. 4g,h. As both MoS_2_ and GQDs are semiconductors with sizable bandgaps, it is important to establish the relative energy levels to determine the band bending and carrier transfer direction when they interact with each other. According to previous reports^10^,^36^,^37^,^38^, the electron affinity of few-layered MoS_2_ is approximately 3.9 eV, which is larger than that of GQDs (2.9 eV)^39^. Because our multilayer MoS_2_ film possesses 2–3 atomic layers, we assume its bandgap is close to that of the monolayer MoS_2_ (1.8 eV)^10^ and much smaller than the bandgap of GQDs (4.5 eV)^39^. Furthermore because these two materials are both *n*-type doped, we propose an energy diagram before the formation of the heterojunction (Fig. 4g). It is clear that the Fermi level of GQDs is closer to the vacuum level than the Fermi level in MoS_2_. When MoS_2_ and the GQDs contact and form a van der Waals heterostructure, the formation of the Fermi level between these materials leads to an injection of electrons from the GQDs to MoS_2_, which is evidenced by the left shift of the threshold voltage in the electrical transfer curve shown in Fig. 4b. The movement of electrons causes the band bending^36^, as shown in Fig. 4h, which manifests as a small energy barrier.

The light-matter interaction in the MoS_2_-GQDs heterojunction is complicated, as both of these two materials strongly absorb and emit light. It is proposed that at least five photoelectrical processes are involved, as schematically illustrated in Fig. 4h. According to the steady state PL measurements (Fig. S7), it is notable that the PL of the GQDs is almost quenched whereas the PL of MoS_2_ is maintained. We suggest that a re-absorption of emitted photons (process II) from the GQDs by MoS_2_ is the origin of the PL quenching. Furthermore, this process will induce new electron-hole pairs, which in return serve as charge carriers to increase the photocurrent. Moreover, the photoexcited electrons in the valence band of the GQDs may be injected into MoS_2_ through a tunnelling process (process III), which will also contribute extra electrons to increase the photocurrent. The time-resolved PL decay transients show that the decay time of MoS_2_-GQDs is decreased to 85 ps, which is much shorter than the decay times of MoS_2_ (224 ps) and GQDs (337 ps) (Fig. S7). This indicates that the recombination rate is faster in MoS_2_-GQDs, which results from the transfer of holes from GQDs to MoS_2_ (process IV) and is stimulated by an endogenous electrical field. Consequently, the photoresponse is faster in MoS_2_-GQDs photodetectors, as evidenced in Fig. 4d.

## Conclusions

In summary, highly responsive photodetectors based on the hybrid of MoS_2_ and GQDs have been demonstrated, which have a photoresponsivity of 1.6 × 10^4^ AW^−1^ and a photogain of 2.4 × 10^7^. The incorporation of GQDs into MoS_2_ forms an *n-n* type heterostucture with significantly improved carrier mobility on account of the injection of electrons from the GQDs to MoS_2_. The improved photodetection performance is attributed to enhanced light-matter interactions resulting from the tunnelling of photoexcited carriers from the GQDs to MoS_2_, the re-absorption of emitted photons from the GQDs by MoS_2_ and the transfer of holes from the GQDs to MoS_2_. Our work demonstrates an effective and applicable way to optimise the photodetection performance of MoS_2_ and other analogous two-dimensional TMD materials.

## Methods

### Growth of the MoS_2_ films and GQDs

The MoS_2_ films were grown directly on a SiO_2_/Si substrate in a 2-inch quartz tube. High purity MoO_3_ powder (99.5%, Alfa Aesar) and sulphur (99.5%, Sinopharm Chemical Reagent Shanghai Co., Ltd) were used as the precursors. The sulphur powder was placed outside the hot zone and mildly sublimated by heating belts at 140 °C. The distance between the sulphur and MoO_3_ was approximately 25 cm. The SiO_2_/Si substrates were placed downstream along the MoO_3_ source. Argon (99.999%) was used as the carrier gas to convey MoO_3–x_ onto the SiO_2_/Si substrates. The tube was flushed with argon to maintain normal pressure. The growth conditions were 700 °C for 30 min with an argon gas flow rate of 50 standard-state cubic centimetres per minute (sccm). The GQDs were synthesised following a previously reported methodology^25^.

### Characterisation of the MoS_2_ films

The MoS_2_ films were wet-transferred onto a quartz substrate by etching away the SiO_2_ in a potassium hydroxide solution for subsequent UV-visible spectral measurements on a UV-visible-infrared spectrometer (PerkinElmer, Lambda750). Raman and PL spectra were measured on a micro-Raman system (Horiba Jobin Yvon, LabRAM HR 800) with a 514 nm excitation laser. The Raman and PL images were obtained with a confocal micro-Raman system (WITec, Alpha 300a) with a 532 nm laser. The spatial resolution is approximately 250 nm with a focused laser by a 100× objective lens. The time-resolved PL decay transients were measured with a transient state fluorescence spectrometer (HORIBA Jobin Yvon, FL-TCSPC). Chemical composition analysis was performed using XPS (KRATOS Analytical, AXIS Ultra DLD). The surface morphology of the samples was examined by SEM (FEI, Quanta 200FEG) and AFM (Bruker, Dimension Icon). The microstructures of MoS_2_ and GQDs were investigated using HRTEM (FEI, Tecnai G2 F20). For the preparation of the TEM samples, the MoS_2_ film was also wet-transferred onto TEM grids.

### Fabrication of the phototransistors

The phototransistors were fabricated directly on SiO_2_/Si substrates after material growth. The SiO_2_/Si substrate consists of an oxidisation layer (thickness: 300 nm) and highly doped *n*-type silicon, which serves as the back gate electrode. A typical device fabrication process involves UV lithography to define the device pattern and electron-beam evaporation to deposit the source and drain electrodes (*i.e.*, 100 nm Au on top of 5 nm Ti). The GQDs solution was drop-casted onto the MoS_2_ phototransistor device followed by a gentle heat treatment at 70 °C for 15 min.

### Photoelectrical measurements

Photoelectrical measurements were performed on a probe station (Cascade M150) equipped with a semiconductor property analyser (Keithley 2400) at room temperature in ambient conditions. A tapered optical fibre connected with laser diodes (405 nm, 532 nm and 635 nm) was used to capture the illumination of the photodetector devices. The area size of the laser illumination is much larger than the detection area of the devices (~70000 μm^2^). The excitation light power mentioned in this work is referred to the actual light power shinning onto the device.

## Additional Information

**How to cite this article**: Chen, C. *et al.* Highly responsive MoS_2_ photodetectors enhanced by graphene quantum dots. *Sci. Rep.* **5**, 11830; doi: 10.1038/srep11830 (2015).

## Supplementary Material

Supplementary Information
