# Supplementary material for: Highly responsive MoS2 photodetectors enhanced by graphene quantum dots
Source: Sci Rep. 2015 Jul 3;5:11830. doi: 10.1038/srep11830 (PMC4490346; doi:10.1038/srep11830)
Supplement: Supplementary Information [file srep11830-s1.pdf]

**Supporting Information for**

# Highly responsive $MoS_2$ photodetectors enhanced by graphene quantum dots

Caiyun Chen[1,‖], Hong Qiao[1,‖], Shenghuang Lin[1,2,‖], Chi Man Luk[2], Yan Liu[1], Zaiquan Xu[3], Jingchao Song[3], Yunzhou Xue[1], Delong Li[4], Jian Yuan[1], Wenzhi Yu[1], Chunxu Pan[4], Shu Ping Lau[2], Qiaoliang Bao[1,3,*]

[1]Institute of Functional Nano and Soft Materials (FUNSOM), Jiangsu Key Laboratory for Carbon-Based Functional Materials and Devices, and Collaborative Innovation Center of Suzhou Nano Science and Technology, Soochow University, Suzhou 215123, P. R. China

[2]Department of Applied Physics, The Hong Kong Polytechnic University, Hung Hom, Hong Kong SAR, China

[3]Department of Materials Engineering, Monash University, Clayton, VIC 3800, Australia

[4]School of Physical and Technology, Wuhan University, Wuhan 430072, P. R. China

[‖]These authors contributed equally to this work.

Correspondence and requests for materials should be addressed to Q. B. (email: qlbao@suda.edu.cn)

**Supplementary Figures**

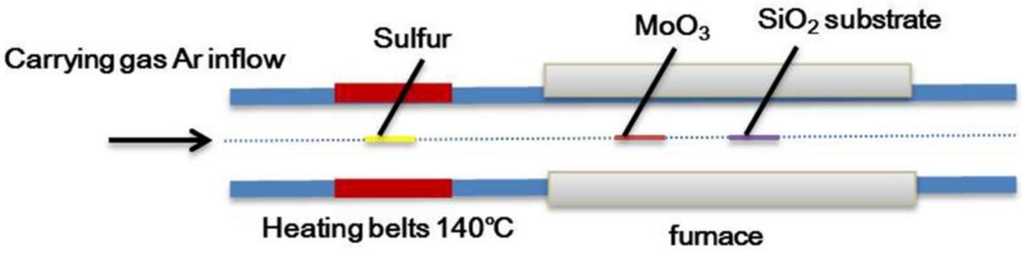


**Supplementary Figure S1. Experimental setup for the synthesis of multilayer $MoS_2$ film on $SiO_2$/Si substrate.** The system mainly consists of three parts: furnace, quartz tube and heating belts.

The experimental setup to synthesize $MoS_2$ on $SiO_2$/Si substrate is depicted in supplementary Fig. S1. This chemical vapor deposition (CVD) setup mainly includes the heating zone within the furnace, the heating belts and a 2-inch quartz tube where the source materials (*e.g.*, sulphur, $MoO_3$) and substrates are placed. The distance between sulphur powder and $MoO_3$ powder is about 25 cm. The pressure is maintained at ambient pressure by flushing Ar gas with a flow rate of 50 sccm.

The furnace temperature is first increased from room temperature to 500 ℃ within 30 min with a faster heating rate of ~16 ℃/min. Following the sample area was heated from 500 ℃ to 700 ℃ with a slower heating rate of 6 ℃/min and then kept at 700 ℃ for 30 min. At the same time, sulphur is mildly sublimated at 140 ℃ by the heating belts. During this period, the $MoO_3$ powder was partially reduced to volatile suboxide species of $MoO_{3-x}$ by sulphur, and then conveyed by carrier gas to the $SiO_2$ substrate to form $MoS_2$ film. Finally, the furnace was allowed to cool down to room temperature naturally.

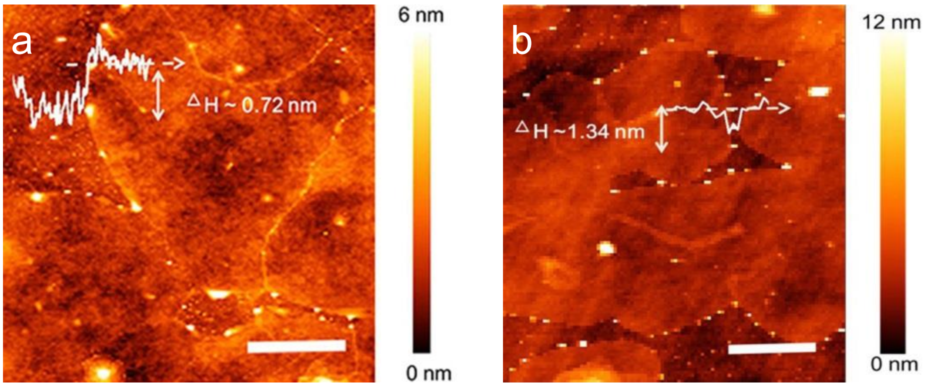


**Supplementary Figure S2. AFM images for $MoS_2$ thin films with different thickness.** (a) Monolayer $MoS_2$, scale bar: 500 nm. (b) Bilayer $MoS_2$, scale bar: 1 μm.

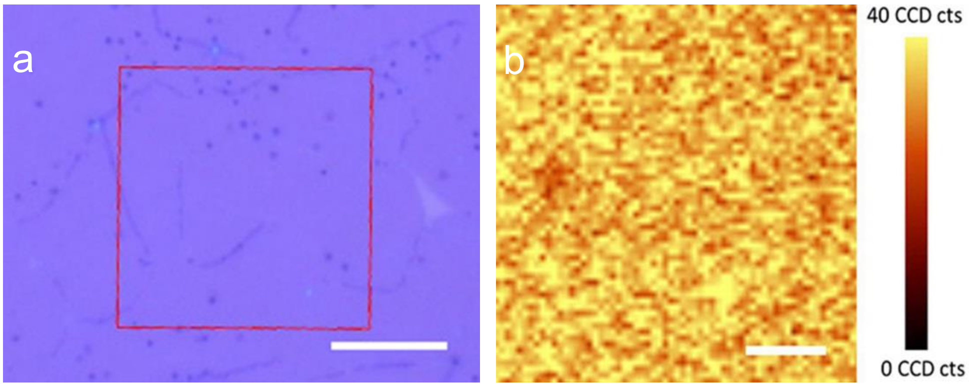


**Supplementary Figure S3. Optical and Raman images of $MoS_2$ film.** (a) Optical image of $MoS_2$ film area. Scale bar: 10 μm. The Raman image shown in the main text was scanning in the area indicated by red box. (b) Corresponding Raman image of $MoS_2$ film integrated from 350 to 390 $cm^{-1}$. Scale bar: 4 μm.

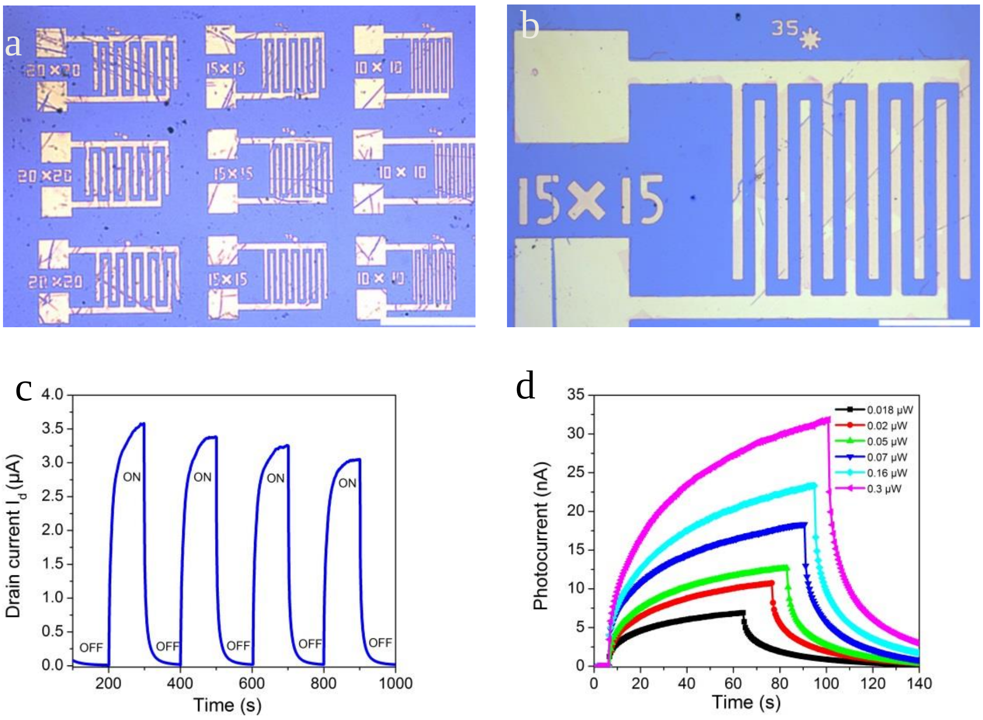


**Supplementary Figure S4. Optical images and performance of $MoS_2$ phototransistors.** (a) Optical image of $MoS_2$ phototransistors array. The scale bar is 500 μm. (b) The optical image of single $MoS_2$ phototransistor device. The scale bar is 100 μm. (c) Time-dependent drain current showing the optical ON-OFF switching ($V_{ds}$ = 1 V, $V_g$ = 0 V) under the excitation of 405 nm laser. (d) Time-dependent photocurrent under different laser powers. $V_{ds}$ = 1 V, $V_g$ = 0 V. Laser wavelength: 405 nm.

In order to characterize the photodetection performance of the thin film $MoS_2$ devices and hybrid $MoS_2$-GQDs devices, we also performed a series of optical-electronic measurements at different light wavelengths. The photoresponse of $MoS_2$ photodetector under the excitation of 532 nm is shown in supplementary Fig. S5. The photoresponse of $MoS_2$-GQDs photodetector under the excitation of 635 nm light is shown in supplementary Fig. S6.

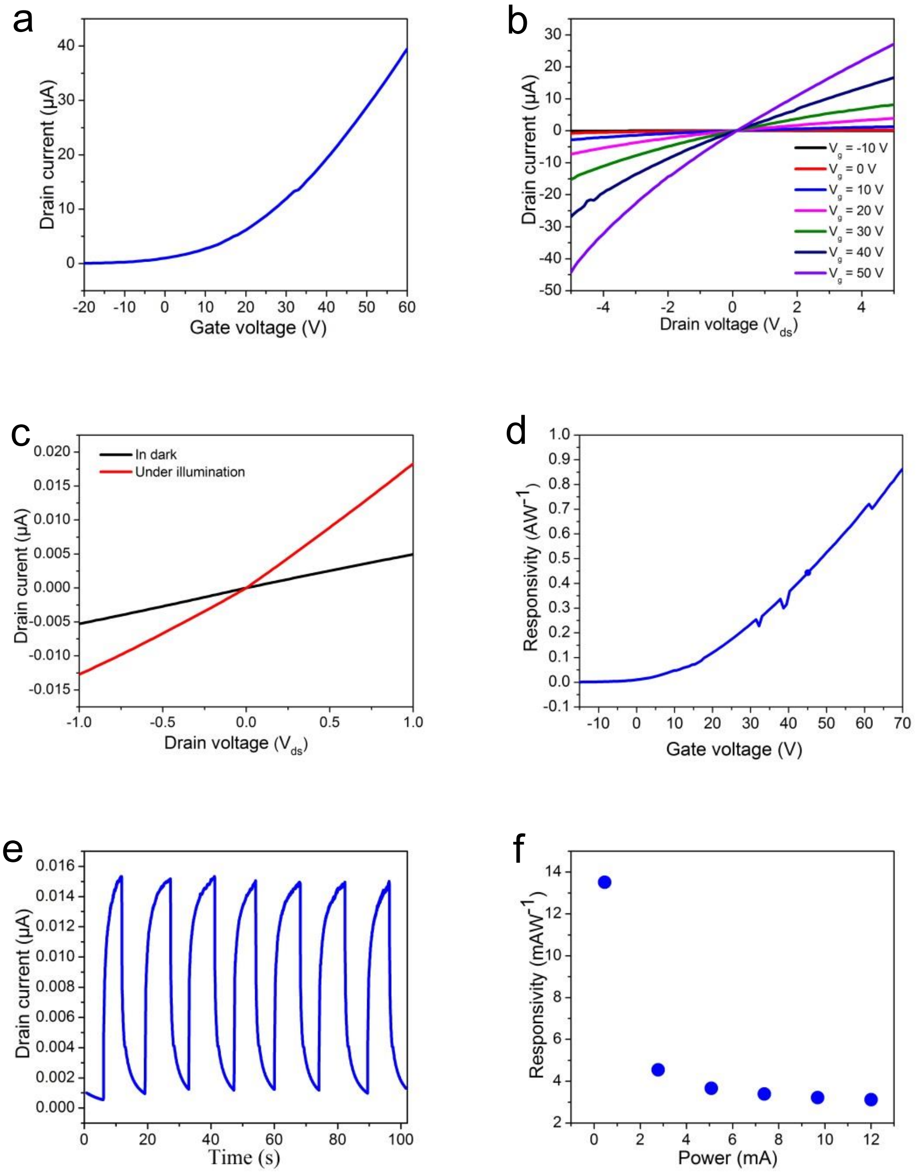


**Supplementary Figure S5. Results of $MoS_2$ phototransistors**. (a) Transport properties of $MoS_2$ transistor. The transport curve represents the drift of drain current when gate voltage is swept from -20 V to 60 V and the source-drain voltage is fixed at 1 V. (b) Drain current as a function of drain voltage at different gate bias. (c) The

drain current without and with light illumination when there is no gate bias. The excitation light is at 532 nm with a power of 0.24 μW. (d) The photoresponsivity versus back gate voltage. Source-drain bias: $V_{ds}$ = 1.5 V; light power: 0.24 μW. (e) Time-dependent photoresponse. $V_{ds}$ = 1 V, $V_g$ = 0 V. (f) The dependence of optical responsivity on light power. $V_{ds}$ = 1 V, $V_g$ = 0 V.

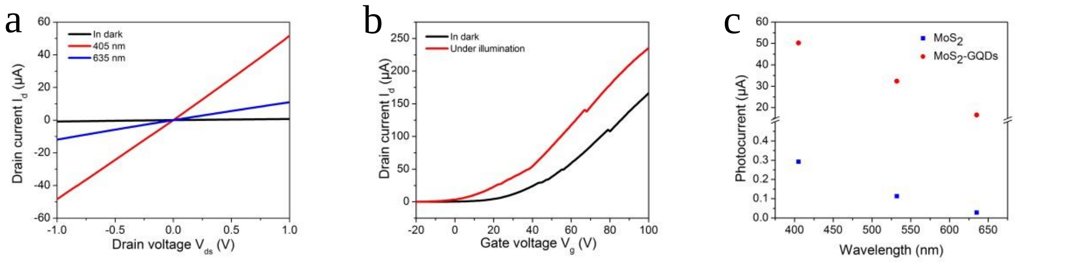


**Supplementary Figure S6. Results of $MoS_2$-GQDs phototransistors**. (a) *I*-*V* characterisitcs of $MoS_2$-GQDs photodetector in dark and at excitation of 405 nm and 635 nm light. (b) Device drain current as a function of gate voltage ($V_{ds}$ = 1 V) in dark and under illumination of 635 nm. (c) The dependence of device photocurrent on wavelength (405 nm, 532 nm and 635 nm) with a power of 1.74 μW.

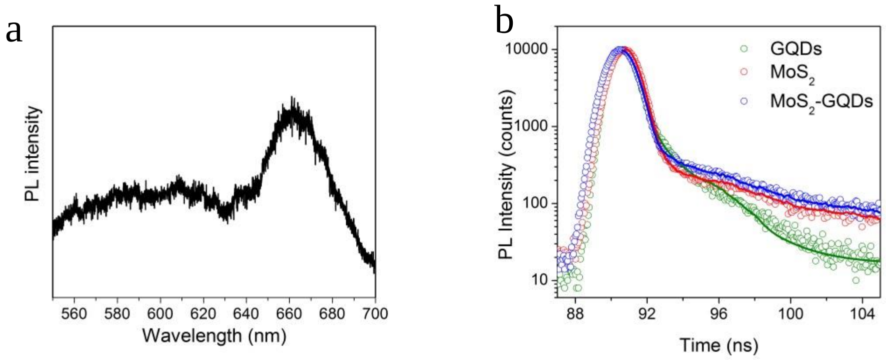


**Supplementary Figure S7. PL and lifetime of $MoS_2$/GQDs.** (a) PL spectrum of $MoS_2$-GQDs on $SiO_2$/Si substrate. (b) Time-resolved PL decay transients measured at 561 nm for $MoS_2$, GQDs and $MoS_2$-GQDs .
